# Stigma, access to healthcare, and HIV risks among men who sell sex to men in Nigeria

**DOI:** 10.7448/IAS.20.01.21489

**Published:** 2017-04-20

**Authors:** Trevor A Crowell, Babajide Keshinro, Stefan D Baral, Sheree R Schwartz, Shauna Stahlman, Rebecca G Nowak, Sylvia Adebajo, William A Blattner, Manhattan E Charurat, Julie A Ake, for the TRUST/RV368 Study Group

**Affiliations:** ^a^ U.S. Military HIV Research Program, Walter Reed Army Institute of Research, Silver Spring, MD, USA; ^b^ Henry M. Jackson Foundation for the Advancement of Military Medicine, Bethesda, MD, USA; ^c^ Walter Reed Program-Nigeria, Abuja, Nigeria; ^d^ Department of Epidemiology, Johns Hopkins Bloomberg School of Public Health, Baltimore, MD, USA; ^e^ Institute of Human Virology, University of Maryland, Baltimore, MD, USA; ^f^ Population Council Nigeria, Abuja, Nigeria

**Keywords:** men who have sex with men, sex work, sub-Saharan Africa, stigma, HIV, epidemiology

## Abstract

**Introduction**: Among men who have sex with men (MSM), men who sell sex (MSS) may be subject to increased sexual behaviour-related stigma that affects uptake of healthcare and risk of sexually transmitted infections (STIs). The objectives of this study were to characterize stigma, access to care, and prevalence of HIV among MSS in Nigeria.

**Methods**: Respondent-driven sampling was used to recruit MSM in Abuja and Lagos into the ongoing TRUST/RV368 study, which provides HIV testing and treatment. Detailed behavioural data were collected by trained interviewers. MSS were identified by self-report of receiving goods or money in exchange for sex with men. Poisson regression with robust error variance was used to explore the impact of sex-selling on the risk of HIV.

**Results**: From 12 initial seed participants, 1552 men were recruited from March 2013-March 2016. Of these, 735 (47.4%) reported sex-selling. Compared to other MSM, MSS were younger (median 22 vs. 24 years, *p* < 0.001) and more likely to identify as gay/homosexual (42.4% vs. 31.5%, *p* < 0.001). MSS were more likely to report perceived and experienced stigmas such as healthcare avoidance (27.6% vs. 21.5%, *p* = 0.005) and verbal harassment (39.2% vs. 26.8%, *p* < 0.001). Total HIV prevalence was 53.4%. After controlling for other factors, HIV prevalence among MSS was similar to that observed among other MSM (relative risk 0.94 [95% confidence interval 0.84–1.05]).

**Conclusions**: These data highlight increased sexual behaviour-related stigma affecting MSS, as compared with other MSM, that limits uptake of healthcare services. The distinct characteristics and risks among MSS suggest the need for specific interventions to optimize linkage to HIV prevention and treatment services in Nigeria.

## Introduction

Despite overall declines in HIV incidence worldwide, concentrated sub-epidemics of HIV have been identified among men who have sex with men (MSM) in most countries where this HIV acquisition risk has been studied [1,2]. Globally, MSM are more than 19 times as likely to be living with HIV than are other reproductive age adults [3]. Stigma, discrimination, and criminalization of same-sex practices potentiate HIV risks among MSM by creating barriers to engagement in healthcare, restricting access to HIV prevention materials such as condoms and condom-compatible lubricants, limiting educational outreach to this key population, and impeding treatment of HIV-infected MSM [4–9]. Within this context, men who sell sex (MSS) may be subject to enhanced sexual behaviour-related stigma, which is defined as stigma that is experienced, perceived, or anticipated as a result of having sex with men. This could alter their risk for acquisition of HIV and other sexually transmitted infections (STIs) [10].

Nigeria is sub-Saharan Africa’s most populous country and has recently increased punitive legislation focused on same-sex practices. This country is currently experiencing the second largest epidemic of HIV in the world, with an estimated 9% of all persons living with HIV globally residing in Nigeria and 10% of new infections occurring in the country, trailing only South Africa in the magnitude of these numbers [11]. In 2013, 14% of global AIDS-related deaths occurred in Nigeria – more than anywhere else in the world [11]. Nigerian MSM experience a disproportionate burden of infections, with reported HIV prevalence as high as 44–66% [12].

MSS predominantly sell sex to male partners, but they may not self-identify as gay or homosexual and frequently maintain sexual relationships with female partners [10,13]. Consequently, their sexual networks tend to be large and non-dense; both network characteristics are associated with increased risk of HIV and other STI transmission. It is therefore critical to understand the burden of HIV and barriers to engagement in evidence-based HIV prevention, treatment, and care services that exist among MSS, particularly in countries where the pace of the HIV epidemic has failed to slow. In this study, we characterize social stigma, perceived and actual barriers to healthcare, and the prevalence of HIV, chlamydia, and gonorrhoea among MSS attending MSM-focused community health centres in Abuja and Lagos, Nigeria.

## Methods

### Study population

Data for these analyses were collected cross-sectionally upon entry into the ongoing TRUST/RV368 cohort study, which enrols MSM participants in Abuja and Lagos, Nigeria, using respondent-driven sampling (RDS) as previously described [14,15]. Briefly, the recruitment process began with identification of several initial study participants, called “seeds.” These seeds were selected to represent a variety of ages, income levels, and neighbourhoods within each city. Initially, three seeds were “activated” at each clinical site to begin recruitment and additional seeds were activated later to facilitate further recruitment as the study progressed. When activated, each seed was given three coupons to distribute to other potential participants in the study. Each new participant in the study was given another three coupons to distribute and waves of recruitment continued in this manner. Incentives were provided for participation in study visits (Naira 2000–3400, equal to about US$6–11, depending on visit) and for referrals (Naira 1500, equal to about US$5). The amounts of these incentives were calculated to equal the costs of time, transportation, and telecommunications associated with study activities. Each participant enrolled into the study was an adult male (over 16 years old in Abuja or over 18 years old in Lagos) who presented with a valid RDS coupon and reported receptive or insertive anal intercourse with a male partner at least once in the 12 months prior to enrolment. This method of recruitment has been shown to reach highly marginalized populations of MSM representing appropriate candidates for HIV prevention and treatment services [15,16].

Upon enrolment, each participant underwent testing for HIV and other STIs. Trained interviewers administered, in either English or Hausa, a standardized questionnaire to collect demographic and behavioural data, including detailed information about sexual activities, perceived stigma, and healthcare engagement. A study physician performed a complete medical examination and recorded the participant’s medical history. These baseline evaluations were split over two study visits approximately two weeks apart.

Participants who enrolled in the TRUST/RV368 study between 20 March 2013 and 31 March 2016 and answered the interviewer’s question about sex-selling were included in these analyses. Seed participants were included if they had been activated to begin recruitment and these data were available. All participants provided written informed consent prior to enrolment. The study protocol was approved by institutional review boards at the Nigerian Federal Capital Territory and Nigerian Ministry of Defense, Abuja, Nigeria; the University of Maryland, Baltimore, MD, USA; and the Walter Reed Army Institute of Research, Silver Spring, MD, USA.

### Definition of “men who sell sex”

For these analyses, “men who sell sex” were those who reported one or more partners in response to the following question: “Thinking about when you had sex with any men in the last 12 months, how many men did you have anal or oral sex with in exchange for things you wanted or needed such as money, drugs, food, shelter or transportation?” Participants who reported zero partners in response to this question were categorized as “men who do not sell sex.” Participants who did not answer this question were excluded from these analyses.

### Assessment of sexual behaviour stigma

Experiences of sexual behaviour stigma were ascertained by self-report with questions designed to evaluate several types of sexual behaviour stigma, including perceived, experienced, and anticipated [17–22]. Perceived stigma refers to an individual’s awareness of negative societal attitudes that results in feelings such as fear or shame. Experienced stigma includes open acts of discrimination, such as denial of services, harassment, or violence. Anticipated stigma is the fear or expectation of discrimination [23,24]. Differential burden of sexual behaviour stigma was assessed among participants categorized as MSS as compared to other MSM in the study.

The specific questions used to assess stigma have been previously described [25]. Briefly, in separate questions, participants were asked whether they have ever felt afraid to seek healthcare or walk around in public places because they have sex with men. Participants were also asked whether they have ever been denied healthcare, verbally harassed, or blackmailed because they have sex with men or whether they have ever been physically assaulted or forced to have sex for any reason. Responses were recorded on paper case report forms and imported into a research database using the TeleForm (Hewlett-Packard Inc., Palo Alto, CA, USA) data capture system. A trained data verifier confirmed the accuracy of each data capture.

### Testing for HIV, chlamydia, and gonorrhoea

Each participant was screened for HIV infection using fingerstick blood specimens for parallel testing according to national guidelines with Determine® (Alere, Watham, MA, USA) and Uni-gold® (Trinity Biotech, Co Wicklow, Ireland) kits [26]. Voided urine and self- or physician-collected rectal swabs were tested for *Chlamydia trachomatis* and *Neisseria gonorrhoea* using the ultra-sensitive Aptima Combo 2® assay (Hologic, Bedford, MA, USA) assay. All testing was conducted according to package inserts.

### Statistical analyses

Comparisons between MSS and men who do not sell sex were identified a priori as the primary interest of these analyses. Across these two groups, demographic characteristics and behavioural risk factors of interest were compared using Pearson’s chi-squared test for categorical variables, Student’s *t*-test for continuous variables with a normal distribution, and Wilcoxon rank-sum test for continuous variables with a skewed distribution. Unadjusted and adjusted Poisson regression models with robust error variance were used to estimate the relative risk (RR) with 95% confidence intervals (CIs) associated with selling of sex and various outcomes of interest, including prevalent HIV infection and other STIs [27]. Multivariable models estimated the independent effects of compensated sex, age, gender identity, sexual orientation, religion, education, occupation, location and marital status.

The primary analyses used pooled data from two independent populations that were recruited using RDS to evaluate internal relationships between sex-selling and HIV/STI prevalence. The primary analyses did not account for any sampling bias introduced through the RDS recruitment methodology. Sensitivity analyses were conducted using two different methods to account for this potential source of bias. First, models were run with generalized estimating equations clustered on seed. Second, models were run with weighting of variables using the Volz–Heckathorn RDS estimator [28].

For all analyses, missing data were carried backward from the scheduled 3-month follow-up study visit if available or categorized as “unknown” if unavailable. A two-sided type I error of 5% was considered statistically significant. All analyses were performed using Stata 13.0 (StataCorp LP, College Station, TX, USA).

## Results

### Study population

Participants were recruited from 12 seeds, including five seeds in Abuja with up to 27 waves of accrual and seven seeds in Lagos with up to 24 waves of accrual. One of the seeds in Lagos did not yield any referrals.

From March 2013 to March 2016, 1592 men enrolled in the TRUST/RV368 study. Of these, 1552 answered the baseline question about sex-selling and are included in these analyses. The study population comprised 946 participants in Abuja and 606 in Lagos (Table 1). Overall, 735 of 1552 (47.4%) reported selling sex to at least one male partner during the preceding year.

**Table 1.** Study population characteristics

**Characteristics**	**Overall** **(*****n***** = 1552)**	**Men who sell sex** (***n***** = 735)**	**Men who do not sell sex (*****n***** = 817)**	***p*****-value**
**Age**				
Median (IQR)	23 (20–27)	22 (20–25)	24 (21–28)	<0.001
≤21 years	554 (35.7)	320 (43.5)	234 (28.6)	<0.001
22–30 years	847 (54.6)	383 (52.1)	464 (56.8)	
>30 years	151 (9.7)	32 (4.4)	119 (14.6)	
**Gender identity**				
Male	1263 (81.4)	565 (76.9)	698 (85.4)	<0.001
Female	186 (12.0)	118 (16.1)	68 (8.3)	
Other/Unknown	103 (6.6)	52 (7.1)	51 (6.2)	
**Sexual orientation**				
Gay/Homosexual	569 (36.7)	312 (42.4)	257 (31.5)	<0.001
Bisexual	971 (62.6)	417 (56.7)	554 (67.8)	
Other/Unknown	12 (0.8)	6 (0.8)	6 (0.7)	
**Religion**				
Christian	1107 (71.3)	498 (67.8)	609 (74.5)	0.002
Muslim	433 (27.9)	234 (31.8)	199 (24.4)	
None/Other/Unknown	12 (0.8)	3 (0.4)	9 (1.1)	
**Education level**				
Junior Secondary or Less	257 (16.6)	156 (21.2)	101 (12.4)	<0.001
Senior Secondary	810 (52.2)	423 (57.6)	387 (47.4)	
Higher than Senior Secondary	472 (30.4)	153 (20.8)	319 (39.0)	
Unknown	13 (0.8)	3 (0.4)	10 (1.2)	
**Occupation**				
Unemployed	319 (20.6)	178 (24.2)	141 (17.3)	<0.001
Student	368 (23.7)	172 (23.4)	196 (24.0)	
Professional/Self-Employed	387 (24.9)	156 (21.2)	231 (28.3)	
Entertainment/Hospitality	198 (12.8)	111 (15.1)	87 (10.6)	
Driver/Labourer	41 (2.6)	19 (2.6)	22 (2.7)	
Other/Unknown	239 (15.4)	99 (13.5)	140 (17.1)	
**City**				
Abuja	946 (61.0)	428 (58.2)	518 (63.4)	0.037
Lagos	606 (39.0)	307 (41.8)	299 (36.6)	
**Marital status**				
Single/Never Married	1362 (87.8)	664 (90.3)	698 (85.4)	0.005
Married/Living with a woman	112 (7.2)	35 (4.8)	77 (9.4)	
Living with a man	28 (1.8)	12 (1.6)	16 (2.0)	
Divorced/Widowed/Separated/Other	50 (3.2)	24 (3.3)	26 (3.2)	
**Children**				
No	1400 (90.2)	681 (92.7)	719 (88.0)	0.005
Yes	150 (9.7)	54 (7.3)	96 (11.8)	
Unknown	2 (0.1)	0 (0.0)	2 (0.2)	
**Injection drug use**				
No	1508 (97.2)	710 (96.6)	798 (97.7)	0.022
Yes	37 (2.4)	24 (3.3)	13 (1.6)	
Unknown	7 (0.5)	1 (0.1)	6 (0.7)	
**Non-Injection drug use**				
No	1147 (73.9)	521 (70.9)	626 (76.6)	0.010
Yes	397 (25.6)	212 (28.8)	185 (22.6)	
Unknown	8 (0.5)	2 (0.3)	6 (0.7)	

IQR, interquartile range. All data are presented as *n* (%) unless otherwise specified. *P*-values were calculated using Student’s *t*-test for age as a continuous variable and Pearson’s chi-squared test for all other variables.

Compared to men who do not sell sex, MSS tended to be younger (median 22 vs. 24 years, *p* < 0.001), more likely to self-identify as female (16.1% vs. 8.3%, *p* < 0.001), more likely to self-identify as gay/homosexual (42.4% vs. 31.5%, *p* < 0.001), and less likely to have progressed beyond secondary education (20.8% vs. 39.0%, *p* < 0.001). Although injection drug use (IDU) was uncommon overall, MSS were twice as like to report ever injecting drugs (3.3% vs. 1.6%, *p* = 0.022) and were also more likely to report non-injection drug use (28.8% vs. 22.6%, *p* = 0.010).

### Sexual behaviours

MSS reported a median of three male partners who gave payment for sex in the preceding 12 months, with an interquartile range (IQR) of 2–6 partners. The maximum number of paying male partners reported was 350. In addition to receiving payment for sex, 233 of 735 MSS (31.7%) reported giving payment to another male partner in exchange for sex within the preceding 12 months, compared to 204 of 817 men who do not sell sex (25.0%, *p* = 0.002). Among MSS, the median number of compensated and uncompensated male anal sex partners in the preceding 12 months was 7 (IQR 4–12), compared to 4 (IQR 2–8) among men who do not sell sex (*p* < 0.001).

**Figure 1.**
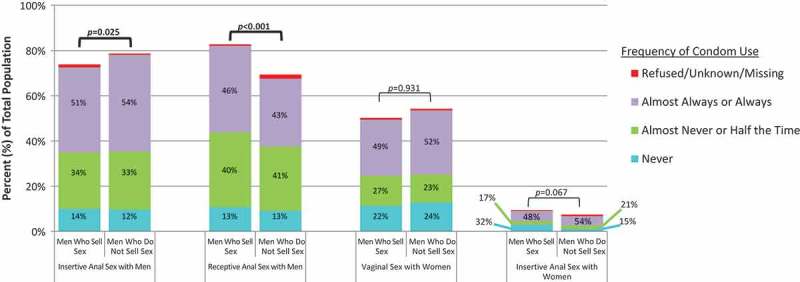
Sexual behaviours and condom use. Bar height represents the percentage of all participants who reported each sexual behaviour within the 12 months prior to enrolment. Pearson’s chi-squared test was used to compare the proportion of participants reporting each sexual behaviour between men who sell sex and men who do not sell sex. Statistically significant *p*-values (*p* ≤ 0.05) are shown in bold. Shaded areas represent the percentage of participants who reported each frequency of condom use during a sexual behaviour out of all participants who reported that behaviour. There were no statistically significant differences in the frequency of condom use between men who sell sex and men who do not sell sex.

Compared to other MSM, MSS were less likely to report insertive anal sex with men (73.9% vs. 78.7%, *p* = 0.025) and more likely to practice receptive anal sex (82.7% vs. 69.4%, *p* < 0.001, Figure 1). Anal sex with women was uncommon among study participants, but there was a trend towards this practice being slightly more common among MSS as compared to men who do not sell sex (9.4% vs. 7.5%, *p* = 0.067). Self-reported use of condoms did not vary significantly between MSS and other MSM for any of the sexual behaviours examined.

### Stigma and access to care

**Figure 2.**
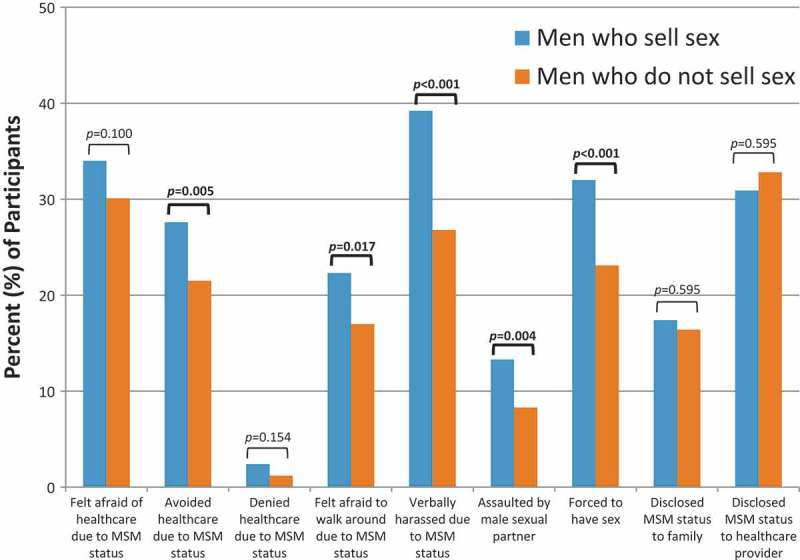
Stigma and access to care among men who sell sex and men who do not sell sex. Bars represent the percentage of all study participants who reported each indicator of stigma during the baseline structured interview. Comparisons are presented between men who sell sex and men who do not sell sex, with statistically significant *p*-values (*p* ≤ 0.05) shown in **bold**.

MSS were more likely than men who do not sell sex to have avoided healthcare because they have sex with men (27.6% vs. 21.5%, *p* = 0.005, Figure 2). Few participants had ever been denied healthcare services because they have sex with men and no difference in the denial of services was observed between MSS and men who do not sell sex (2.4% vs. 1.2%, *p* = 0.154). MSS were more likely than other MSM to have ever felt afraid to walk around in public because they have sex with men (22.3% vs. 17.0%, *p* = 0.017), more likely

to have been verbally harassed because they have sex with men (39.2% vs. 26.8%, *p* < 0.001), more likely to have been assaulted by a male sexual partner (13.3% vs. 8.3%, *p* = 0.004), and more likely to have been forced to have sex (32.0% vs. 23.1%, *p* < 0.001). No differences were noted in rates of disclosing MSM status to family (17.4% vs. 16.4%, *p* = 0.595) or healthcare providers (30.9% vs. 32.8%, *p* = 0.474).

### HIV and other STIs

Among 1,170 participants with HIV screening results available, 625 (53.4%) were seropositive. The percentage of participants without available HIV testing results did not differ between MSS and men who do not sell sex (25.0% vs. 24.2%, *p* = 0.715).

The crude prevalence of HIV was marginally lower among MSS as compared to men who do not sell sex (50.6% vs. 55.9%, *p* = 0.072, Table 2). However, after adjusting for other factors, no association was observed between selling sex and prevalent HIV infection (RR 0.94 [95% CI 0.84–1.05]) as compared to men who do not sell sex (Table 3). This observation was robust to sensitivity analyses evaluating the association between selling sex and prevalent HIV infection in multivariate models that used generalized estimating equations to account for clustering emanating from the same seed participant (RR 0.94 [95% CI 0.79–1.11]) and multivariate models that used weighting of variables according to RDS variance estimators (RR 0.88 [95% CI 0.73–1.06]).

**Table 2.** Prevalence of sexually transmitted infections

Sexually transmitted infection	Men who sell sex	Men who do not sell sex	*p*-value
**HIV** (*n* = 1170)	279/551 (50.6%)	346/619 (55.9%)	0.072
**Chlamydia**			
Urogenital (*n* = 927)	17/418 (4.1%)	30/509 (5.9%)	0.207
Rectal (*n* = 921)	68/413 (16.5%)	68/508 (13.4%)	0.287
**Gonorrhoea**			
Urogenital (*n* = 929)	17/419 (4.1%)	17/510 (3.3%)	0.559
Rectal (*n* = 928)	112/417 (26.9%)	100/511 (19.6%)	**0.031**

Positive screening results for each sexually transmitted infection are shown. The denominator for percentage calculations is the total number of participants screened for that particular infection. Individual participants may not have been screened for every infection for reasons such as participant refusal, insufficient specimen collection, or changes to the protocol schedule of evaluations after study initiation. Statistical comparisons were made using Pearson’s chi-squared test and significant *p*-values (≤0.05) are shown in **bold**.

**Table 3.** Risk factors for HIV infection

Characteristics	Unadjusted risk ratio (95% CI)	Adjusted risk ratio (95% CI)
**Compensated sex**		
Men who do not sell sex	Reference	Reference
Men who sell sex	0.90 (0.81–1.01)	0.94 (0.84–1.05)
**Age**		
≤21 years	Reference	Reference
22–30 years	**1.34 (1.18–1.53)**	**1.33 (1.16–1.52)**
>30 years	**1.39 (1.15–1.66)**	**1.40 (1.14–1.72)**
**Gender identity**		
Male	Reference	Reference
Female	**1.42 (1.26–1.61)**	**1.40 (1.23–1.59)**
Other/Unknown	**1.27 (1.07–1.51)**	**1.22 (1.02–1.45)**
**Sexual orientation**		
Gay/Homosexual	Reference	Reference
Bisexual	**0.89 (0.80–0.99)**	0.94 (0.84–1.05)
Other/Unknown	0.63 (0.29–1.38)	0.48 (0.18–1.25)
**Religion**		
Christian	Reference	Reference
Muslim	**0.77 (0.67–0.89)**	0.87 (0.75–1.02)
None/Other/Unknown	0.88 (0.50–1.56)	0.93 (0.47–1.84)
**Education level**		
Junior Secondary or Less	Reference	Reference
Senior Secondary	**1.48 (1.18–1.87)**	1.23 (0.98–1.55)
Higher than Senior Secondary	**1.55 (1.22–1.97)**	**1.26 (1.00–1.60)**
Unknown	**1.81 (1.15–2.86)**	**1.95 (1.31–2.91)**
**Occupation**		
Unemployed	Reference	Reference
Student	**0.83 (0.70–0.97)**	0.87 (0.74–1.02)
Professional/Self-Employed	0.88 (0.75–1.02)	0.89 (0.76–1.04)
Entertainment/Hospitality	0.83 (0.68–1.01)	0.86 (0.71–1.04)
Driver/Labourer	1.00 (0.73–1.39)	1.01 (0.72–1.41)
Other/Unknown	1.05 (0.90–1.23)	0.96 (0.82–1.13)
**City**		
Abuja	Reference	Reference
Lagos	**1.28 (1.15–1.43)**	**1.25 (1.11–1.40)**
**Marital status**		
Single/Never Married	Reference	Reference
Married/Living with a woman	1.08 (0.88–1.34)	1.23 (0.99–1.54)
Living with a man	**1.32 (1.01–1.71)**	1.12 (0.84–1.49)
Divorced/Widowed/Separated/Other	**1.12 (0.87–1.45)**	1.07 (0.83–1.38)

CI, confidence interval. Poisson regression models with robust error variance were used to model factors associated with prevalent HIV infection. The adjusted model included all listed factors. Statistically significant *p*-values (≤0.05) are shown in **bold**.

Prior awareness of HIV status did not differ between MSS and men who do not sell sex who had positive HIV tests upon enrolment (49.5% vs. 51.5%, *p* = 0.389).

Chlamydia and gonorrhoea were each more common at the rectal site than at the urogenital site. The crude prevalence of rectal gonorrhoea was higher among MSS (26.9% vs. 19.6%, *p* = 0.031) but this bivariate association was not significant after adjusting for age, gender identity, sexual orientation, religion, education, occupation, location and marital status (RR 1.03 [95% CI 0.72–1.47]). No statistically significant associations were observed between MSS status and urogenital gonorrhoea, rectal chlamydia, or urogenital chlamydia.

## Discussion

Selling of sex was reported by about half of participants, making it a common phenomenon within this population of Nigerian MSM. Although MSS may include participants who engaged in sexual-economic exchanges that they did not consider commercial in nature, this number is consistent with wide-ranging previous reports of commercial or compensated sex among MSM across Sub-Saharan Africa including 33–74% of MSM in Kenya [29–31], 59–84% in Tanzania [32,33], 32–44% in Uganda [34,35], 22% in Senegal [36], 29% in Cote d’Ivoire [37], and 24–55% in Nigeria [38–40]. In this study, MSS reported a small number of paying partners per year, suggesting that these may not have been exclusively commercial interactions. More likely, this is part of a social environment in which sexual exchange may be facilitated by money [41]. Prior research indicates that few men who sell sex to other men recognize this practice as sex work [10]. Within our study, MSS appear to represent a distinct subgroup of MSM with different demographic characteristics, sexual behaviours, and experiences of stigma.

While the majority of MSM in this study considered themselves to be bisexual, a larger proportion of MSS identified as gay/homosexual as compared to other MSM. Notably, no participants self-identified as heterosexual, which differs from prior studies conducted in other settings which have suggested that MSS are more likely than other MSM to self-identify as heterosexual [42–45]. This could reflect a unique characteristic of the MSS population in Nigeria or this finding may be influenced by the study methodology, which was conducted at MSM-focused community health centres and used an RDS-based system of participant referrals for recruitment. Heterosexual-identified MSS may be less likely than other MSM to engage in a study associated with being MSM or may belong to different social networks than the participants in this study, which would preclude involvement.

There was a high burden of HIV and rectal STIs among participants in this study. After adjusting for other factors, no differences were observed in the prevalence of HIV, chlamydia or gonorrhoea between MSS and other MSM. Although no difference in the frequency of condom use was observed between MSS and other MSM in this study, previous investigation has found condom use to be higher among MSM when engaged in sex work than in other sexual encounters [46,47]. A potentially increased risk of rectal STIs among MSS due to a higher frequency of anal receptive intercourse may have been mitigated by an increased frequency of condom use while engaged in sex-selling, although overall condom-use frequency did not vary between groups and many study participants reported condomless sex. Prior research has suggested that condom use is more common during sexual encounters with financial motivations than during sexual encounters driven by emotion [41,47]. In the context of a population with a high prevalence of untreated and undertreated HIV infections, this suggests the potential for addressing barriers to the uptake of evidence-based and rights-affirming HIV prevention strategies as an effective intervention to reduce the risk of HIV and other STIs. Such interventions should include packages of condom-compatible lubricants [48–50], a choice of proven barrier methods [51–53], and scale up of oral pre-exposure prophylaxis (PrEP) [54].

There appears to be enhanced stigma related to same-sex practices among MSS in this study. This is in addition to the well-described stigma that may occur because of HIV status and sexual orientation [55–57]. MSS were more likely to report fear, verbal harassment, assault and sexual violence than were other MSM. These amplified experiences of stigma may contribute to the observed avoidance of healthcare among MSS. Interventions to reduce or circumvent healthcare avoidance should be pursued, such as treatment support [58–60] and HIV self-testing [61,62]. Actual denial of healthcare was uncommon, which may be due in part to the avoidance of healthcare due to perceived stigma, limited disclosure of same-sex practices to clinicians, and utilization of trusted community-based venues such as the recruitment sites for this study. Other measures of experienced stigma, such as verbal harassment and assault, were more common among MSS than among men who do not sell sex. Some of these differences may have been attributable to greater visibility of sexual preferences among MSS as compared to other MSM, due to factors such as a greater prevalence of female gender identity among MSS or factors associated with attracting customers for compensated sex [10,63]. Compounded stigma and perceived barriers to healthcare may limit uptake among MSS as compared to other MSM.

There are several strength and limitations of the analyses reported here. The use of the RDS recruitment strategy for this study enabled recruitment and characterization of a highly marginalized population of Nigerian MSM. Administration of standardized questionnaires and structured interviews allowed for the gathering of detailed information about sexual behaviours, condom use, stigma, and other population characteristics. However, self-reporting of sensitive information may result in some inaccuracies, particularly given the stigma surrounding same-sex practices in Nigeria. To optimize honest reporting of sexual practices, this study was conducted in close partnership with MSM-focused healthcare centres serving two large, urban populations. Supporting the assumption of minimal reporting biases, no participants self-identified as heterosexual, even though prior studies in other settings have suggested that many MSS do not consider themselves gay/homosexual or bisexual [42–45]. Findings of this study may not be generalizable to MSM communities in other locations or to MSS who self-identify as heterosexual. Further limitations in generalizability may have been introduced by sampling bias inherent in the RDS recruitment methodology, although consistency in statistical inferences across multiple models designed to account for this non-random recruitment method offers reassurance that our observations are valid.

## Conclusions

This study reveals important differences between MSS and other MSM, including different demographic characteristics, sexual behaviours, and experiences of stigma. Although no significant differences were observed in the prevalence of HIV and other STIs in this study population with a high burden of infection, a distinct need for intervention supporting MSS is apparent. This population experiences compounded stigmas and demonstrates greater avoidance of healthcare than is observed among men who do not sell sex, suggesting the need for decentralized HIV prevention and treatment approaches providing HIV self-testing, PrEP, and HIV and STI treatment support. These data highlight the potential individual and population benefits for male sex workers and other MSS of specific interventions to improve access to, and the ultimate impact of, HIV prevention and treatment services in Nigeria.
